# Preparation and Characteristics of SiO_x_ Coated Carbon Nanotubes with High Surface Area

**DOI:** 10.3390/nano2020206

**Published:** 2012-06-18

**Authors:** Aeran Kim, Seongyop Lim, Dong-Hyun Peck, Sang-Kyung Kim, Byungrok Lee, Doohwan Jung

**Affiliations:** 1 Fuel Cell Research Center, Korea Institute of Energy Research, Daejeon 305-343, Korea; Email: aeran6608@naver.com (A.K.); dhpeck@kier.re.kr (D.-H.P.); ksk@kier.re.kr (S.-K.K.); brlee@kier.re.kr (B.L.); doohwan@kier.re.kr (D.J.); 2 Advanced Energy Technology, University of Science & Technology, Daejeon 305-350, Korea

**Keywords:** carbon nanotubes, silicon oxide, hybrid materials, porosity

## Abstract

An easy method to synthesize SiO_x_ coated carbon nanotubes (SiO_x_-CNT) through thermal decomposition of polycarbomethylsilane adsorbed on the surface of CNTs is reported. Physical properties of SiO_x_-CNT samples depending on various Si contents and synthesis conditions are examined by X-ray diffraction (XRD), X-ray photoelectron spectroscopy (XPS), nitrogen isotherm, scanning electron microscope (SEM), and transmission electron microscope (TEM). Morphology of the SiO_x_-CNT appears to be perfectly identical to that of the pristine CNT. It is confirmed that SiO_x_ is formed in a thin layer of approximately 1 nm thickness over the surface of CNTs. The specific surface area is significantly increased by the coating, because thin layer of SiO_x_ is highly porous. The surface properties such as porosity and thickness of SiO_x_ layers are found to be controlled by SiO_x_ contents and heat treatment conditions. The preparation method in this study is to provide useful nano-hybrid composite materials with multi-functional surface properties.

## 1. Introduction

Hybrid materials combining carbon nanotubes (CNT) with the oxides have been synthesized to obtain improved or sometimes new functions in various applications. For example, TiO_2_-CNT hybrids in electrocatalysis have been reported to increase catalytic activity of platinum and its CO-tolerance for alcohol electro-oxidation [1]. In gas sensors, SnO_2_-CNT hybrids improved response and recovery by combining instinct electrical properties of SnO_2_ and excellent adsorption properties of CNT having high surface area [2,3,4,5,6], and ZnO-CNT hybrids shown higher photocatalytic activity than ZnO bulk material by combining advantages of ZnO, such as high optical activity and high sensitivity for UV-Vis light, and advantages of CNT, such as high mechanical strength and chemical stability [7]. In addition, the oxide-CNT hybrids were applied in supercapacitors (MnO_2_) and field emission (MgO) [8,9]. SiO_2_-CNT hybrids have been prepared for various applications such as oxidation resistance, optic applications, biosensor and catalytic applications due to their high surface area and versatility of silica chemistry [10,11,12].

Generally, activation of carbons is to increase porosity and attach functional groups over their surface through partly destroying their structure. Especially, CNT, which has the basal surface (less reactive than the graphitic edges), is required to be activated in harsh conditions such as chemical oxidation using strong acid such as HNO_3_, H_2_SO_4_, and KMnO_4_ [13,14,15,16,17]. Chemical oxidation is a very effective way to increase the solubility and chemical reactivity, as well as the porosity and surface area [18,19]. However, the defects formed by such treatments can lessen the superior mechanical and electronic properties of CNT [20].

In this study, a surface modification was carried out through coating SiO_x_ over CNT, which might be an efficient way to functionalize the CNT surface without making structural defects. SiO_x_-CNT composites were synthesized using a simple method with adsorption of hydrophobic silicon containing polymer on the CNT surfaces and then thermal decomposition under prescribed conditions.

## 2. Experimental Section

### 2.1. Chemicals

The CNT sample used in this study is a multi-wall type (diameter 10–40 nm, purity 95%), purchased from CNT Co., Ltd. Polycarbomethylsilane (MW~800) was purchased from Sigma-Aldrich Co., and tetrahydrofuran (THF) was obtained from SAMCHUN chemicals. 

### 2.2. Preparation of SiOx-CNT

**Figure 1:**
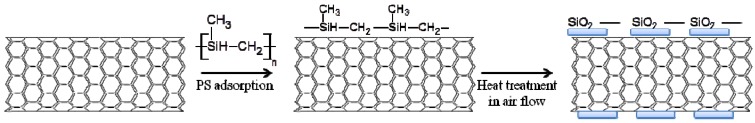
Schematic diagram of the SiO_x_ coated carbon nanotubes (SiO_x_-CNT) preparation by Polycarbomethylsilane (PS) coating and heat treatment.

Figure 1 shows schematically the preparation process of SiO_x_-CNT samples. Polycarbomethylsilane (PS), which is a silicon-containing polymer, was used as the SiO_x_ precursor. PS of 1 g was completely dissolved in 150 mL THF under vigorous stirring at room temperature for 30 min. CNTs were added into the solution, which was stirred for 15 h. The mixtures were shortly sonicated, and then the solvent was removed, using a rotary evaporator under reduced pressure at 35 °C, and the mixture powder was dried in an oven at 80 °C over 12 h. 

SiO_x_-CNT was obtained through thermal decomposition of PS-CNT mixture. The heat treatment carried out at different temperature (300 °C, 400 °C, 500 °C, and 700 °C) in an air atmosphere fed at a rate of 200 cm^3^/min. Heating rate was 5 °C/min and at set point temperatures maintained for 2 h.

The names of the samples were defined as SCm0n (m: ratio of CNTs, n: the hundred digit of heat treatment temperatures). The ratio (weight) of CNT to PS is fixed to “n:1”. For example, “SC103” is prepared from the precursor ratio of CNT to PS, 1:1 (w/w) at 300 °C. 

### 2.3. Characterization

Thermal gravimetric analysis (TGA) was examined, using STA409PC (NETZSCH). The temperature range was from 25 °C to 900 °C with a constant heating rate of 5 °C/min in air flow. 

The morphology of samples was characterized by SEM (S-4700, Hitachi) and TEM (Tecnai F20, Philips). For TEM measurement, very small quantity of the samples was dispersed in ethanol solution and the solution was sonicated for 5 min. The well-dispersed suspension was dropped onto a TEM grid. 

The specific surface areas and pore parameters were obtained by analyzing the nitrogen adsorption-desorption isotherms recorded at 77 K using a surface area analyzer (BELSORP-mini, BEL JAPAN INC.). Prior to this measurement, the samples were degassed at 250 °C for 3 h. The pore size was analyzed from the desorption isotherm using the BJH (Barrett-Joyner-Halenda) method. 

XRD (RINT2000, Rigaku) was carried out to investigate the graphitic structure of CNT before and after SiO_x_ coating with heat treatment at various temperatures. Scans were done for 2θ values between 10 °C and 90 °C at 5 °C/min. To inspect chemical states of silicon and carbon in SiO_x_-CNT composites, XPS (AXIS NOVA, KRATOS) was also performed.

## 3. Results and Discussion

### 3.1. Morphology of SiO_x_-CNT Composites

**Figure 2:**
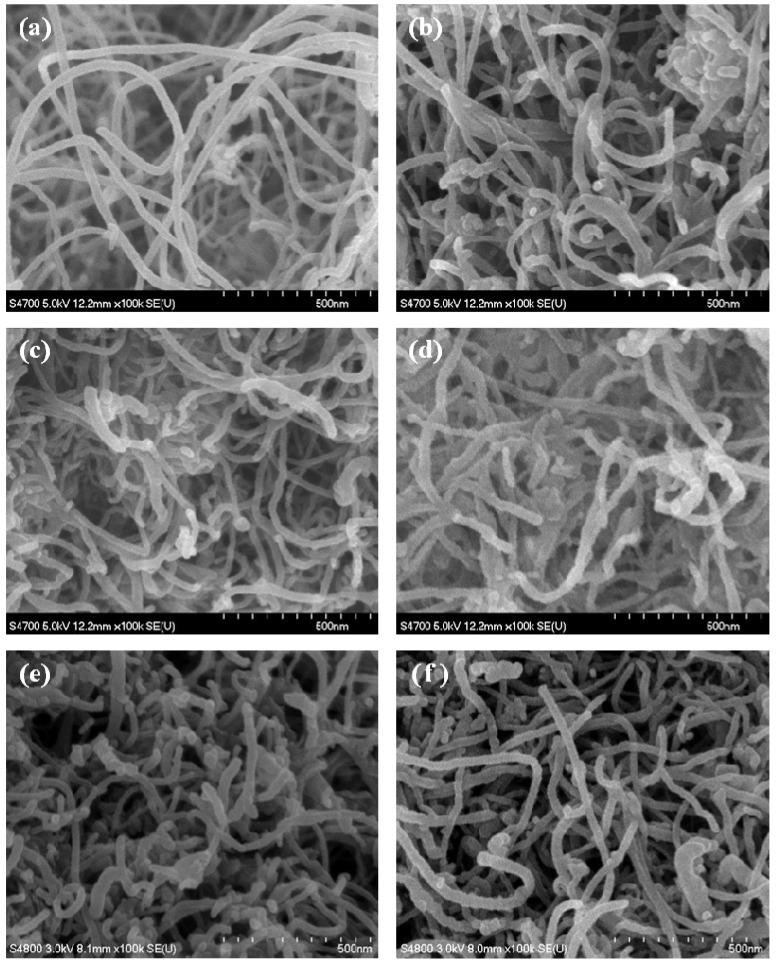
SEM images of (**a**) pristine CNTs, (**b**) SC104, (**c**) SC105, (**d**) SC107, (**e**) SC204, and (**f**) SC304.

Figure 2 shows SEM images of CNTs and various SiO_x_-CNT samples. Figure 2a shows an image of pristine CNTs, and clearly indicates a structural feature of CNTs having thin tube shape. As shown in Figure 2b,c,e,f, the morphology of SiO_x_-CNT samples appear to be almost similar with the pristine CNT, despite of SiO_x_ coating. When CNT was completely combusted at 700 °C in air (sample SC107), appearance of the sample was also almost the same as pristine CNT, and any noticeable silica particulates or aggregates were not found under SEM (Figure 2d). It indicates that PS is uniformly coated over the whole CNT surface, resulting in silica nanotubes with the same dimensions as CNT.

**Figure 3:**
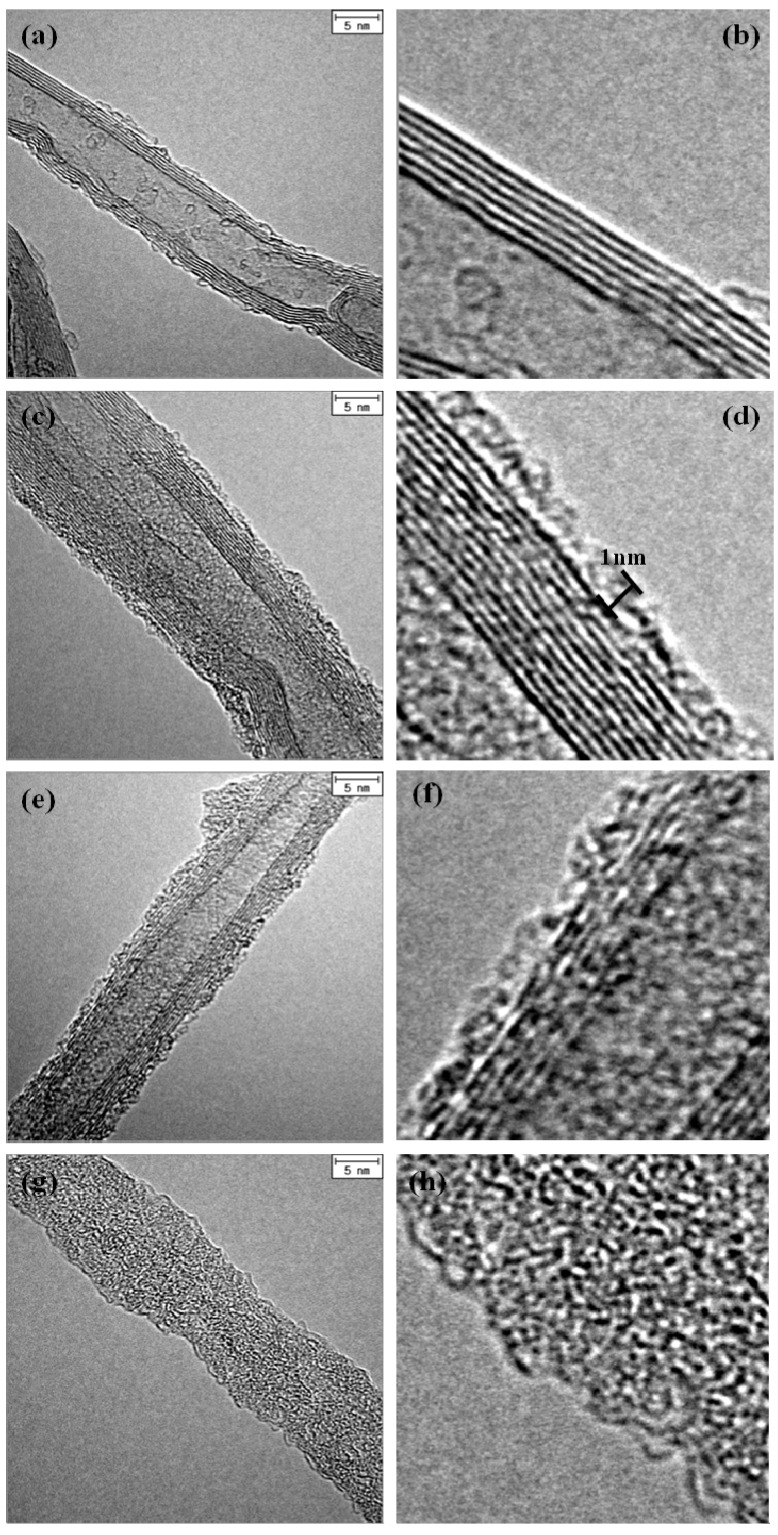
TEM images of (**a,b**) pristine CNTs, (**c,d**) SC103, (**e,f**) SC104, (**g,h**) SC107.

The structure of the samples was observed by TEM, examining SiO_x_ layers on the CNT. Figure 3 shows TEM images of original CNTs and various SiO_x_-CNT samples treated at different temperatures. For the pristine CNT (Figure 3a,b), and several graphitic layers were confirmed to be well arranged in a manner of concentric cylinders with a clean surface overall. In SiO_x_-CNT samples, an additional layer, which has an amorphous structure, is found on the CNT surface, as shown in Figure 3c–f. The amorphous layer spreads over the CNT surface with an approximate thickness of 1 nm, although it is not perfectly uniform. The graphitic layers of CNT in the SiO_x_-CNT samples appear to be straight-aligned and almost intact. Decrease of the image resolution for the graphitic layers in Figure 3d,f is probably due to the SiO_x_ layer over the CNT surface. In Figure 3g,h, SiO_x_ nanotubes with amorphous structure were confirmed, and their geometrical morphology in TEM images appears to be identical to the pristine CNT, as observed in SEM (Figure 2d). 

### 3.2. Surface Properties of SiO_x_-CNT

The sample preparation, specific surface area and pore volume are summarized in Table 1. The contents of CNT and SiO_x_ in these samples were evaluated using TG under an air atmosphere through complete combustion of CNT. Specific surface area and total pore volume were calculated from the nitrogen adsorption/desorption isotherms. The surface area as well as pore volume increases as the temperature increases. In case of the sample SC105, the specific surface area is approximately two times higher than that of the pristine CNT. As the Si content decreases (SC20m and SC30m series), the increase of the specific surface area tends to be reduced, but it shows still higher (1.6 times at least for the samples heat-treated at 500 °C) than that of the pristine. Hence, formation of new porosity must be caused by silicon oxide species coated on CNT, and it can be controlled by the heat treatment conditions and the Si compositions. 

**Table 1:** Surface properties and the contents of SiO_x_ in SiO_x_-CNT samples.

Sample	Content (wt %)		Heat treatment conditions	Specific surface area (m^2^/g)	Total pore volume (cm^3^/g)
	SiOx	CNT			
CNT	0	100	–	198	0.372
SC103	48.8	51.2	300 °C, air, 2h	261	0.199
SC104	48.8	51.2	400 °C, air, 2h	352	0.253
SC105	48.8	51.2	500 °C, air, 2h	405	0.273
SC107	100	0	700 °C, air, 2h	671	0.768
SC203	32.1	67.9	300 °C, air, 2h	214	0.317
SC204	32.1	67.9	400 °C, air, 2h	315	0.388
SC205	32.1	67.9	500 °C, air, 2h	335	0.357
SC303	26.9	23.1	300 °C, air, 2h	220	0.344
SC304	26.9	23.1	400 °C, air, 2h	250	0.384
SC305	26.9	23.1	500 °C, air, 2h	328	0.434

**Figure 4:**
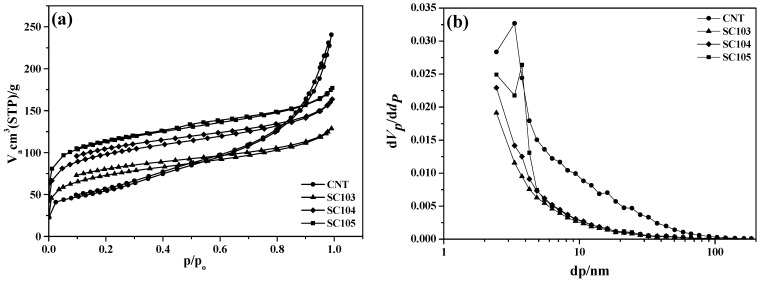
(**a**) Nitrogen isotherm and (**b**) pore size distribution curve of SiO_x_-CNT samples (48.8 wt % SiO_x_) treated at different temperatures (300 °C, 400 °C, 500 °C).

Figure 4 shows nitrogen isotherm profiles and corresponding pore size distribution of pristine CNT and SiO_x_-CNT samples synthesized according to the heat treatment temperatures. From the low relative pressure (below p/p_0_ 0.1), micropores are confirmed to increase, probably originating from silicon species. The increase of porosity according to increase of temperature, might reflect progress of thermal decomposition of PS and at the same time formation of micropores. The adsorption/desorption isotherm profile of the pristine CNT is of the type II in the IUPAC classification, which is as obtained from a non-porous or macroporous adsorbent [21]. For SiO_x_-CNT samples, the type of isotherm profiles is found to change to that of type I, which corresponds to that of microporous solid. Comparing CNT with SC10m series, the total pore volume decreased with the silicon species coating, although the specific surface area increased. Hence, the porosity of SiO_x_-CNT samples must be dominated by silicon species. It is highly-microporous. 

From the pore size distribution (Figure 4b), the level of large pore volume over 10 nm appears to be higher in the pristine CNT than in SiO_x_-CNT samples. The large pore of the CNT sample must originate from inter-space formed in an agglomerate of entangled nanotubes, because their diameters are around 10 nm. The low level of such a large pore volume in SiO_x_-CNT samples indicates that such an agglomeration is disassembled to some degree during the preparation procedure such as coating PS in the solution or heat treatment. The surface coating may be effective for dispersion of CNT, although there is not sufficient proof at present. 

### 3.3. XPS Analysis of the Chemical Status of the SiO_x_-CNT Composites

**Figure 5:**
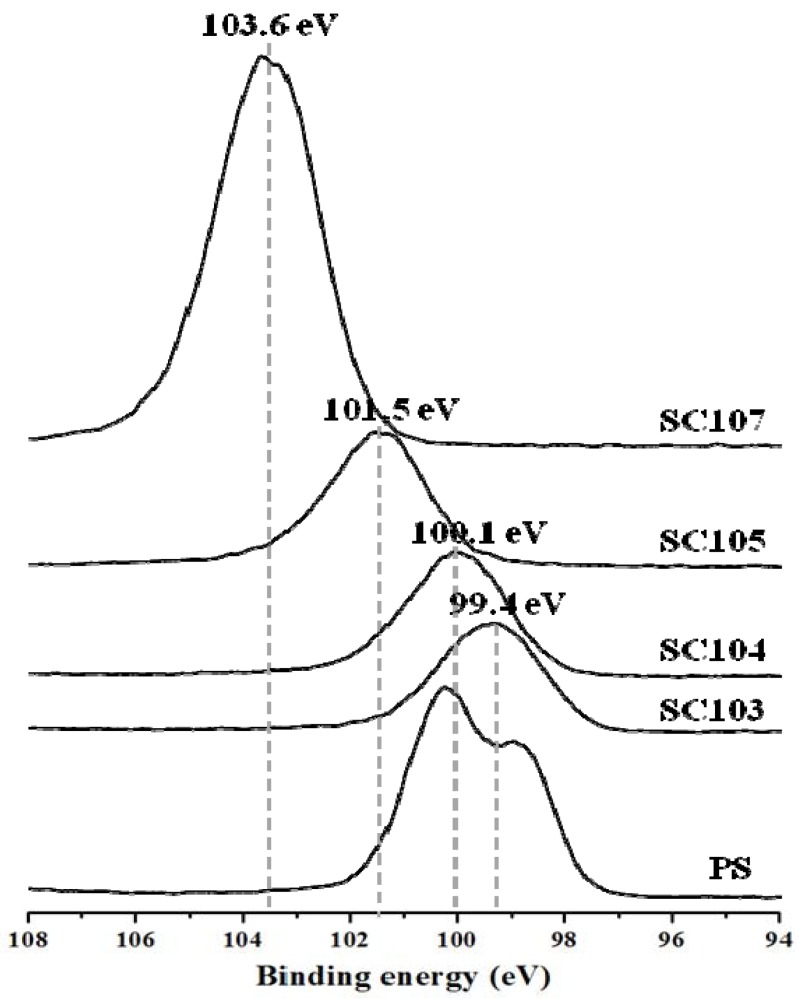
Si-2p XPS profiles of PS and SiO_x_-CNT samples.

**Figure 6:**
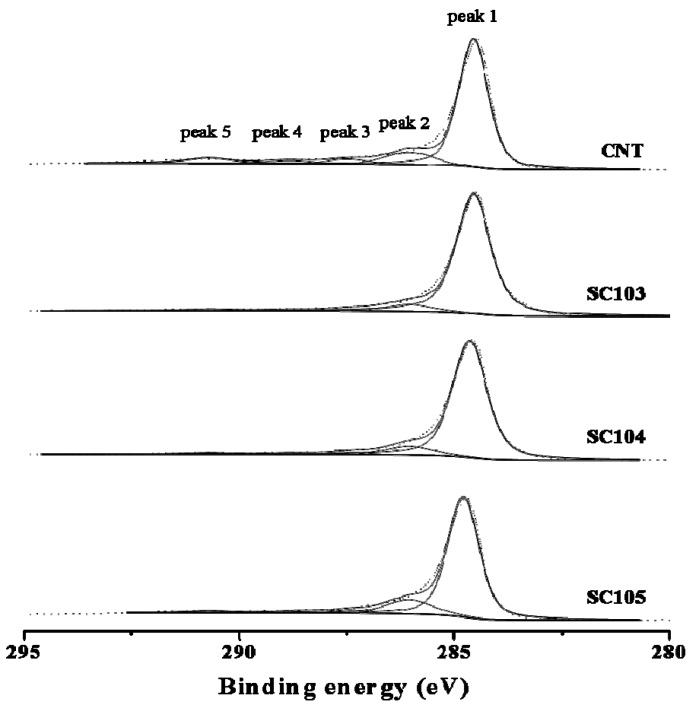
C-1s XPS profiles of CNT and SiO_x_-CNT samples.

XPS analysis of SiO_x_-CNT composites was performed to examine the oxidation state of silicon (Figure 5) and carbon (Figure 6) on the surface. Figure 5 shows Si-2p spectra of PS and SiO_x_-CNT treated at different temperatures. Binding energy of Si 2p indicates the oxidation state of Si atom such as Si^0+^SiH (99.4 eV), Si^1+^ (100.3 eV), Si^2+^ (101.2 eV), Si^3+^ (102.0 eV), and Si^4+^ (103.0 eV) [22]. Si-2p peaks of SiO_x_-CNT were positively shifted from 99.4 eV to 103.5 eV by increasing treatment temperature. Si atom was partly oxidized to SiO_x_ (x < 2) below 500 °C for 2 h, while it was fully oxidized to SiO_2_ at 700 °C. Specifically, the silicon atoms in the sample prepared at 300 °C have still direct bond to alkyl moiety, not yet to be oxidized, and those of SC104 is slightly oxidized, being close to the state of Si^1+^. For the sample prepared at 500 °C, the oxidation number of silicon atoms ranges from 2+ to 3+. Considering the silicon oxidation state with the surface area and porosity (Table 1 and Figure 4), the more oxidation proceeds, the more pores are formed. Hence, micropores of SiO_x_-CNT composites must stem from thermal decomposition of alkyl moieties and rearrangement of silicon species to oxides. 

Figure 6 shows C-1s core level spectra of CNT and SiO_x_-CNT composites, which are deconvoluted to five categories of carbon oxidation states. The peak 1 (284.6 eV) indicates to graphitic carbons, and peak 2 (286.1–286.3 eV) represents carbon in alcohol or ether groups, peak 3 (287.6–287.7 eV) in carbonyl groups, peak 4 (288.6–289.0 eV) in carboxyl or ester groups, and peak 5 (290.6–290.7 eV) in carbonate groups [23]. Profiles of SiO_x_-CNT were rather similar to pristine CNT, which means that the graphitic structure in CNT is almost intact after preparation of SiO_x_-CNT composites. Rather, oxygen functional groups in SC103 and 104 appear to reduce compared to pristine CNT as a result of the decomposition of oxygen functional groups by heat treatment. Whereas, in the case of the SC105 sample, the intensity of peak 2 increases slightly compared to that of CNT, indicating that the CNT surface may be slightly oxidized at 500 °C.

In this study, effects of heat treatment time have yet to be examined, but the oxidation state of silicon atoms is expected to be further controlled by long-time annealing under prescribed conditions. 

### 3.4. XRD Analysis of the SiO_x_-CNT Composites

**Figure 7:**
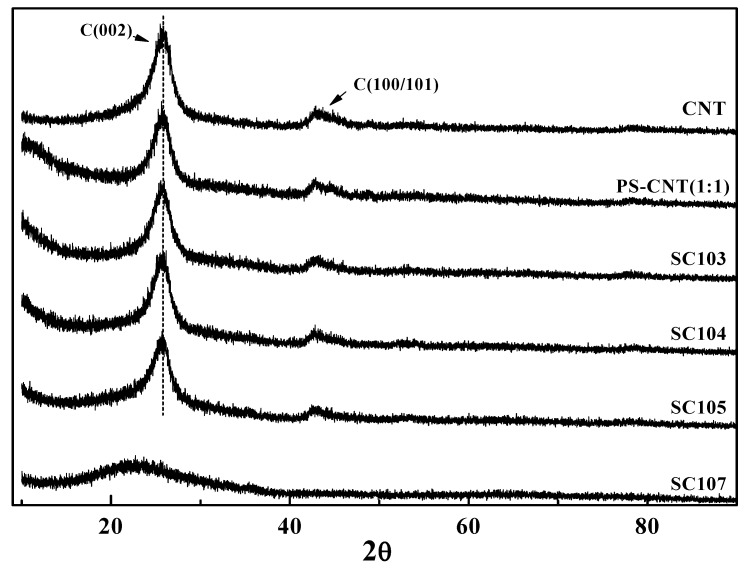
XRD profiles of CNT, PS-CNT and the SiO_x_-CNT according to treatment temperature.

Figure 7 shows the X-ray diffraction profiles of CNTs, PS-CNT and SiO_x_-CNT treated at 300 °C, 400 °C, 500 °C and 700 °C. In an XRD profile of SC107 sample (100 wt % silica), a peak of amorphous silica was observed at around 25° [24]. It was confirmed from these results that amorphous SiO_x_ layers were formed on the CNT surfaces during the heat decomposition and amorphous peak of SiO_x_ overlapped with the peak of carbon (002) phase. XRD profiles of SiO_x_-CNT samples however are similar to those of CNT. It reflects that the CNT structure in SiO_x_-CNT remains intact without structural defects, which is identical to the results of C-1s XPS results (Figure 6).

## 4. Conclusions

SiO_x_-CNT composites, which consist of CNT at the core and silicon oxide species on the surface, were successfully synthesized by a simple method of thermal decomposition of PS-coated CNT. The hydrophobic PS might be effective to spread uniformly over the surface CNT. Morphology of SiO_x_-CNT composites was nearly identical with the pristine CNT, and the thickness of silicon oxide species was about 1 nm. SiO_x_-CNT composites showed higher specific surface area (over double) than the pristine CNT, and their porosity was found to be controlled by selection of heat treatment conditions and Si/C ratio. The CNT structure in the composites appeared to be intact after the preparation, as confirmed by XPS and XRD. Application of the SiO_x_-CNT hybrid materials to electrodes or catalyst supports for polymer electrolyte fuel cells is in progress; anticipating high surface area for dispersion of catalysts, increased oxidation resistance under electrochemically corrosive environment, and also improved metal-support interaction. 
